# Thoracic fluid content (TFC) using electrical cardiometry versus lung ultrasound in the diagnosis of transient tachypnea of newborn

**DOI:** 10.1007/s00431-024-05507-5

**Published:** 2024-03-15

**Authors:** Nagwa Mohamed Abd EL-Fattah, Heba Saied EL-Mahdy, Manal Fathy Hamisa, Ashraf Mohamed Ibrahim

**Affiliations:** 1 Pediatric Department, Faculty of Medicine, Tanta University, El Bahr St., Tanta Qism 2, Tanta, Gharbia Governorate 31527 Egypt; 2 Radiology Department, Faculty of Medicine, Tanta University, Tanta, Egypt

**Keywords:** Transient tachypnea of the newborn, Lung ultrasound, Thoracic fluid content, Electrical cardiometry

## Abstract

This study aimed to evaluate TFC by EC versus lung ultrasound (LUS) findings for diagnosing and follow-up of TTN in late preterm and term neonates. This prospective observational study was conducted on 80 neonates with gestational age ≥ 34 weeks. TTN group included 40 neonates diagnosed with TTN, and no lung disease (NLD) group included 40 neonates without respiratory distress. LUS and EC were performed within the first 24 h of life and repeated after 72 h. There was a statistically significant increase in TFC in TTN group on D1 [48.48 ± 4.86 (1 KOhm^−1^)] compared to NLD group [32.95 ± 4.59 (1 KOhm^−1^)], and then significant decrease in TFC in D3 [34.90 ± 4.42 (1 KOhm^−1^)] compared to D1 in the TTN group. There was a significant positive correlation between both TFC and LUS with Downes’ score, TTN score, and duration of oxygen therapy in the TTN group.

*Conclusion*: Both LUS and TFC by EC provide good bedside tools that could help to diagnose and monitor TTN. TFC showed a good correlation with LUS score and degree of respiratory distress.

**Table:** 

**What is Known:** *• Transient tachypnea of the newborn (TTN) is the most common cause of respiratory distress in newborns.* *• TTN is a diagnosis of exclusion, there are no specific clinical parameters or biomarker has been identified for TTN.*	
**What is New:** *• Thoracic fluid content (TFC) by electrical cardiometry is a new parameter to evaluate lung fluid volume and could help to diagnose and monitor TTN and correlates with lung ultrasound score.*

**What is Known:**

*• Transient tachypnea of the newborn (TTN) is the most common cause of respiratory distress in newborns.*

*• TTN is a diagnosis of exclusion, there are no specific clinical parameters or biomarker has been identified for TTN.*

**What is New:**

*• Thoracic fluid content (TFC) by electrical cardiometry is a new parameter to evaluate lung fluid volume and could help to diagnose and monitor TTN and correlates with lung ultrasound score.*

## Introduction

Transient tachypnea of the newborn (TTN) is the most common cause of respiratory distress in term gestation newborns. TTN results from impaired clearance of the fetal lung fluid after birth. It can lead to admission to the neonatal intensive care unit (NICU), need for respiratory support, unnecessary antibiotics usage, and prolonged hospital stays [1, 2].

Usually, this condition resolves over 24–72 h. TTN is a diagnosis of exclusion, it is primarily diagnosed based on medical history, typical clinical presentation and chest X-ray may show a radiopaque line in the horizontal fissure of the right lung, fluid infiltrate throughout alveoli, and the lungs may appear hyperinflated [3].

Supportive treatment might be sufficient. But, non‐invasive respiratory support may be required to reduce respiratory distress, reduce the work of breathing, improve clearance of lung liquid, and reduce the duration of tachypnea [4].

Lung ultrasound (LUS) has been used in the diagnosis of many types of neonatal and children’s lung diseases, including TTN, respiratory distress syndrome (RDS), meconium aspiration syndrome (MAS), and pneumonia. LUS is an accurate, non-invasive, and reliable tool for diagnosing TTN and is valuable for the early and differential diagnosis of TTN. The most common ultrasonographic features of TTN are double lung point (DLP), interstitial syndromes or white lungs, and pleural line abnormalities [5].

Electrical cardiometry (EC) is a safe, accurate, and reproducible technique for hemodynamic assessment in children and infants [6] and validated for use in neonates [7, 8]. EC measures alteration in thoracic resistance or impedance, using skin electrodes by sending low amplitude, high frequency electrical current through the thorax. EC is able to isolate the changes in impedance created by the circulation, partly due to the change in orientation of the erythrocytes during the cardiac cycle [9].

Now, a new parameter is available, thoracic fluid content (TFC). It is an indicator of total fluid volume. It represents thoracic intravascular, extravascular, and intrapleural fluid content. Larger TFC indicates a higher total thoracic fluid volume and indirect measure of lung congestion and/or hypervolemia. The TFC showed good correlation with extravascular lung water [10, 11]. Bioimpedance is now the only tool to evaluate TFC continuously and noninvasively at the bedside. TFC is measured as the baseline resistance (bioimpedance) to the passage of a small electrical current through all chest tissues, including skeletal muscle, cardiac muscle, lung, chest wall, subcutaneous fat, bone, and fluid [12].

EC is a bedside tool giving the trend of hemodynamic parameters with nomograms to provide normative data to help to distinguish between normal and pathological conditions [13].

Due to ongoing need for more specific markers of TTN, we conducted the current study to evaluate the TFC by EC versus LUS findings in the diagnosis and follow-up of TTN in late preterm and full-term neonates.

## Methods

This was a prospective observational study that was conducted at NICU of Tanta University Hospitals. The study was approved by the local ethical committee of Faculty of Medicine, Tanta University (No. 34987/10/21). The study was registered at www.ClinicalTrials.gov with ID: NCT05538780. Written parental consent was signed before the enrollment. Eighty newborns with gestational age ≥ 34 weeks (calculated from the first day of last menstrual period and using new Ballard score [14]) were included in the study during the period from January 2022 till December 2022. Neonates with gestational age less than 34 weeks, major congenital abnormalities, congenital heart diseases, neonatal sepsis, neonatal pneumonia, or other causes of respiratory distress other than TTN were excluded.

All enrolled neonates were divided into two groups: TTN group included 40 neonates diagnosed with TTN and met the criteria detailed in the Montreux definition for diagnosis of TTN [15] and no lung disease (NLD) group included 40 term neonates with no respiratory distress signs, normal medical history, normal chest clinical examination, and spontaneously breathing in room air with no need for supplemental oxygen [16].

The study was conducted in accordance with the Helsinki Declaration. The manuscript was prepared following STROBE guidelines [17].

### Methods

All neonates were subjected to full history taking, thorough clinical examination including Downes’ scoring [18], TTN clinical score: to assess degree of respiratory distress in neonates with TTN [19] and routine laboratory investigations included complete blood picture, C-reactive protein, liver and renal function tests, random blood sugar, and blood gasses.

### Lung ultrasound (LUS)

LUS was performed on neonates diagnosed with TTN within the first 24 h of life and repeated after 72 h and was also performed on NLD group within the first 24 h of life by single neonatologist using Siemens Acuson X300 ultrasound machine (Siemens Health Care GmbH, Erlangen, Germany) with 13–5 MHz linear transducer, while the baby was quiet.

The LUS score was measured by dividing each lung into 3 areas (upper anterior, lower anterior, and lateral). For each lung area, a 0- to 3-point score was given (total score ranging from 0 to 18). The LUS score encompassed signs typical of TTN [20]. The transducer was placed perpendicular to the ribs and moved from the midline to the lateral side.

The LUS score 0 indicates A-pattern (defined by the presence of the lung sliding and horizontal A-lines, or less than 3 vertical B-lines); 1, B-pattern (defined as the presence of ≥ 3 well-spaced B-lines); 2, severe B-pattern (defined as the presence of crowded and coalescent B-lines with or without consolidations limited to the subpleural space); 3, extended consolidations which are characterized by tissue echogenicity with static or dynamic air bronchograms [21].

### Electrical cardiometry (EC)

Fluid status was shown as thoracic fluid content (TFC), corrected flow time (FTC), and stroke volume variation (SVV) and was measured on all 40 neonates diagnosed with TTN within the first 24 h of life and repeated after 72 h and was also performed on NLD group within the first 24 h of life by single neonatologist using EC, ICON (Osypka Medical GmbH, Berlin, Germany), while the baby was quiet.

After skin disinfection with alcohol, four skin electrodes were placed on the forehead, at the neck below the left ear, at the left midaxillary line and left thigh and values were recorded for 30 s [22, 23].

TFC is derived from the thoracic electrical base impedance (1/base impedance), but its usefulness is to evaluate either pulmonary fluid overload or soft tissue edema. SVV is defined as the percentage of change between the maximal and minimal stroke volumes over a period of 30 s. FTC is a preload indicator and it is the systole time divided by the square root of cardiac cycle time [22].

### Statistical analysis

The sample size calculation was performed using G.power 3.1.9.2 (Universitat Kiel, Germany). The sample size was calculated according to the sensitivity of LUS for determining TTN was 75% and sensitivity of TFC expected to be ranged between 90 and 100% according to a previous study [24]. Based on the following considerations: 0.05 *α* error and 80% power of the study, allocation ratio 1:1. Two cases were added to each group to overcome dropout. Therefore, 40 patients will be allocated in each group.

Statistical analysis was performed with the Statistical Package for the Social Sciences version 20.0 (SPSS Inc., Chicago, IL, USA). Continuous data are presented as mean ± standard deviation or median (interquartile 25–75) for non-normal distributed variables, whereas discrete data are given as absolute values and percentages. Group means of the continuous variables were compared with Student’s *t*-test or Mann–Whitney *U* test when appropriate. Continuous variables were compared between day 3 and day 1 using paired samples *t*-test or Wilcoxon’s rank sum test when appropriate. Categorical variables were compared with chi-squared test, or Fisher exact test, when appropriate. Correlations were assessed using either Pearson’s correlation test or Spearman’s rank test according to the distribution pattern of the variable. A *p* value of < 0.05 was considered statistically significant with a confidence interval of 95%.

## Results

The characteristics of both groups are shown in Table 1. The rate of cesarean section (CS) was significantly higher in TTN group. Downes’ score was 5.15 ± 0.62 and TTN score was 5.70 ± 0.88 in TTN patients. All the cases of TTN received oxygen support in the form of high flow nasal cannula and duration of oxygen therapy was 3.43 ± 0.90 days.

**Table 1:** Neonate characteristics of the studied groups

**Variable**	**TTN group (*n*** **= 40)**	**NLD group (*n*** **= 40)**	***p*** **value**
Gestational age (weeks)	36.40 ± 1.69	36.90 ± 1.72	0.194
Birth weight (kg)	2.78 ± 0.61	2.97 ± 0.57	0.145
Male sex	23 (57.5%)	21 (52.5%)	0.653
Cesarean section	39 (97.5%)	20 (50%)	< 0.001
Antenatal risk factors			
DM	6 (15%)	5 (12.5%)	0.745
HTN	5 (12.5%)	4 (10%)	1.000
PROM	7 (17.5%)	3 (7.5%)	0.176
Antenatal steroids	22 (55%)	23 (57.5%)	0.822
Placental disorders (previa, insufficiency)	5 (12.5%)	3 (7.5%)	0.712
Multiple pregnancies (twins)	4 (10%)	1 (2.5%)	0.359
Others (UTI, chorioamnionitis)	4 (10%)	1 (2.5%)	0.359

*TTN* transient tachypnea of newborn, *NLD* no lung disease, *DM* diabetes mellitus, *HTN* hypertension, *PROM* premature rupture of membranes, *UTI* urinary tract infection

Regarding LUS findings in TTN group, DLP was present in 31 (77. 5%), pleural line abnormalities (pleural line disappearance, thickening, irregularity, or indistinct appearance) were found in all the cases (100%), alveolar interstitial syndrome (AIS) was observed in 34 (85%), while white lung was present only in 6 (15%) in newborns with TTN.

**Fig. 1:**
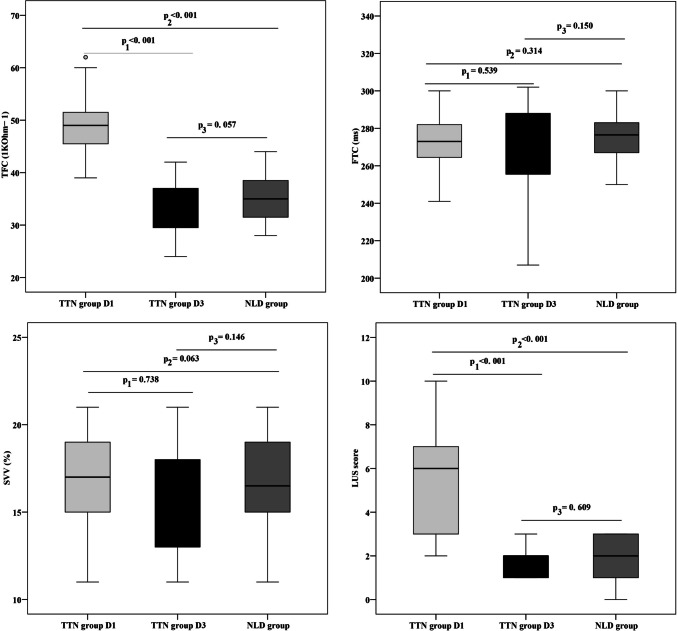
EC parameters (TFC, FTC, and SVV) and LUS score of the studied groups

EC parameters (TFC, FTC, and SVV) and LUS score of the studied groups showed that there was statistically significant increase in TFC and LUS score in TTN group on D1 compared to NLD group. Also, there was statistically significant decrease in TFC in D3 compared to D1 in TTN group. On the other hand, there was no statistically significant difference between TTN group on D3 and NLD group. FTC and SVV showed no statistically significant difference between TTN group on D1 and D3. Also, there was no statistically significant difference between TTN group on D1 and NLD group and between TTN group on D3 and NLD group (Fig. 1).

There was significant positive correlation between LUS score and TFC in the TTN group on D1 and D3. Also, there was significant positive correlation between both TFC and LUS score with Downes’ score, TTN score, and duration of oxygen therapy in the TTN group on D1 and D3, while there was no significant correlation between them and APGAR 1 min, APGAR 5 min, weight, FTC, and SVV in the TTN group on D1 and D3.

## Discussion

TTN is a common cause of respiratory distress in newborns caused by retained fetal lung fluid and consists of a period of rapid breathing that usually resolves within 24–72 h [25]. LUS is a non-invasive tool that is increasingly used in the diagnosis of TTN. It allows visualization of the neonate’s lungs and real-time assessment of conditions like TTN. It also helps exclude other lung diseases and guides further management [26].

Clinical evaluation of cardiac status is important to assess sequelae. EC can be used to noninvasively measure extravascular lung water index in TTN. By tracking changes in thoracic electrical bioimpedance during the cardiac cycle, lung edema and decreased lung water content over time as the condition resolves can be continuously monitored. This technique may allow quantification of disease severity and treatment response without radiation exposure [22].

In the current study, there was no significant difference between both groups as regards gestational age, gender, Apgar 1 and 5 min, anthropometric measurements, or antenatal risk factors, while, as regards mode of delivery, there was a statistically significant increase in CS deliveries in TTN group. This came in agreement with Derbent et al. [27], who showed that the proportion of CS in the TTN group was significantly higher. Another retrospective study by Kasap et al. [28] carried out on 95 newborns with TTN and showed that 79% of the patients were delivered by CS.

TFC was significantly higher in TTN group on D1 compared to NLD group. Furthermore, there was statistically significant decrease in TFC on D3 compared to D1 in TTN group. But there was no statistically significant difference between TTN group on D3 and NLD group. This could be explained by the resorption of lung fluids over time in TTN patients.

In agreement with our findings, Bassiouny et al. [29] revealed that TFC within the first 6 h was high. However, TFC at 24 h of ≤ 24 mL/kg and TFC drop rate at 24 h of > 12% are statistically significant discriminators of TTN from non-TTN.

TFC also was positively correlated with Downes’ and TTN scores in the TTN group. These results agree with Paviotti et al. [12], who found that TFC independently correlates with the presence of respiratory distress at birth and at 24 h of life in late preterm and term newborns.

LUS findings demonstrated DLP only in 31 cases, pleural line abnormalities were found in all the cases, while white lung was present in 6 in newborns with TTN. These results agree with Raimondi et al. [24], who concluded that pleural line with no consolidation is a consistent finding in TTN and the presence of a DLP is not essential for the LUS diagnosis of TTN.

As regards LUS, in our present study, there was significant increase in LUS score in TTN group on D1 compared to NLD group. Also, there was significant decrease in LUS score at D3 compared to D1 in TTN group. The difference between LUS score on D1 and D3 in TTN group could be explained that LUS score decreased progressively over time with resolution of TTN. Our results agreed with Pezza et al. [30], who showed that lung aeration score was evaluated and improved over time in TTN patients.

This also agreed with Li et al. [31], who found that LUS scores decreased significantly from day 1 to day 2. They also found that TTN group exhibited significantly higher LUS scores than did the control group. Also, Yoon et al. [22] demonstrated that LUS score for the prediction of TTN had a sensitivity of 67% and specificity of 97%. This also came in agreement with He et al. [32] meta-analysis which evaluated the diagnostic value of LUS for detecting TTN.

Our data showed a significant positive correlation between LUS and both Downes’ and TTN scores in the studied cases on D1 and D3. This can be explained because LUS score correlated with the severity of respiratory distress which was clinically assessed with Downes’ and clinical TTN scores during the TTN course. This observation came in agreement with the study by Raimondi et al. [24], who found a significant correlation between LUS and Silverman score. Both scores decreased progressively over time.

In our present study, there was a significant positive correlation between LUS score and duration of oxygen therapy. This agreed with Gunes et al. [33], who showed positive correlation between LUS and oxygen exposure. Also, Li et al. [31] observed a moderate correlation between the LUS score and respiratory severity score (RSS), which indicates that the LUS score reflects the clinical respiratory severity of neonates diagnosed with TTN.

We also observed a significant positive correlation between LUS and TFC on the first and third days in TTN cases. This came in agreement with Yoon et al. [22], who found that TFC correlated well with ultrasound in the estimation of extravascular lung fluid. As regards FTC and SVV, there was no statistically significant difference between TTN group and NLD group.

To the best of our knowledge, there are few studies in the current literature evaluating the EC in the diagnosis of TTN. Meanwhile, this is the first study to compare EC versus LUS to diagnose and follow up newborns with TTN.

The current study has some limitations; it was a single-center study, sample size was relatively small, the lack of studies about the reproducibility of the EC in neonates, the time points of evaluation can be variable within first 24 h of age and on the 3rd day, and neonates with different respiratory disorders like RDS were not included.

## Conclusions

Both LUS and TFC by EC provide good bedside tools that could help to diagnose and monitor TTN. TFC showed good correlation with LUS score and degree of respiratory distress. EC has been proposed as a non-invasive, safe, simple, and non-operator dependent real-time monitor for TTN.

## Data Availability

No datasets were generated or analysed during the current study.

## Abbreviations

TTN: Transient tachypnea of the newborn
TFC: Thoracic fluid content
EC: Electrical cardiometry
LUS: Lung ultrasound

## References

[1] Alhassen Z, Vali P, Guglani L, Lakshminrusimha S, Ryan RM (2021). Recent advances in pathophysiology and management of transient tachypnea of newborn. J Perinatol.

[2] Senaldi L, Blatt L, Han JY, Gozum G, Venturini SL, Hauft S (2024). A quality improvement initiative to reduce antibiotic use in transient tachypnea of the newborn. J Perinatol.

[3] Liu J (2014). Lung ultrasonography for the diagnosis of neonatal lung disease. J Matern Fetal Neonatal Med.

[4] Moresco L, Romantsik O, Calevo MG, Bruschettini M (2020). Non-invasive respiratory support for the management of transient tachypnea of the newborn. Cochrane Database Syst Rev.

[5] Liu J, Wang Y, Fu W, Yang CS, Huang JJ (2014). Diagnosis of neonatal transient tachypnea and its differentiation from respiratory distress syndrome using lung ultrasound. Medicine.

[6] Hsu KH, Wu TW, Wang YC, Lim WH, Lee CC, Lien R (2016). Hemodynamic reference for neonates of different age and weight: a pilot study with electrical cardiometry. J Perinatol.

[7] Noori S, Drabu B, Soleymani S, Seri I (2012). Continuous non-invasive cardiac output measurements in the neonate by electrical velocimetry: a comparison with echocardiography. Arch Dis Child Fetal Neonatal Ed.

[8] Gupta D, Dhingra S (2021). Electrocardiometry fluid responsiveness in pediatric septic shock. Indian J Crit Care Med.

[9] Sanders M, Servaas S, Slagt C (2020). Accuracy and precision of non-invasive cardiac output monitoring by electrical cardiometry: a systematic review and meta-analysis. J Clin Monitor Comput.

[10] Michard F (2019). Lung water assessment: from gravimetry to wearables. J Clin Monitor Comput.

[11] Hammad Y, Hasanin A, Elsakka A, Refaie A, Abdelfattah D, Rahman SA (2019). Thoracic fluid content: a novel parameter for detection of pulmonary edema in parturients with preeclampsia. J Clin Monitor Comput.

[12] Paviotti G, De Cunto A, Moressa V, Bettiol C, Demarini S (2017). Thoracic fluid content by electric bioimpedance correlates with respiratory distress in newborns. J Perinatol.

[13] Boet A, Jourdain G, Demontoux S, De Luca D (2016). Stroke volume and cardiac output evaluation by electrical cardiometry: accuracy and reference nomograms in hemodynamically stable preterm neonates. J Perinatol.

[14] Ballard JL, Khoury JC, Wedig KL, Wang L, Eilers-Walsman BL, Lipp R (1991). New Ballard score, expanded to include extremely premature infants. J Pediatr.

[15] De Luca D, van Kaam AH, Tingay DG, Courtney SE, Danhaive O, Carnielli VP (2017). The Montreux definition of neonatal ARDS: biological and clinical background behind the description of a new entity. Lancet Respir Med.

[16] Biasucci DG, Loi B, Centorrino R, Raschetti R, Piastra M, Pisapia L (2022). Ultrasound-assessed lung aeration correlates with respiratory system compliance in adults and neonates with acute hypoxemic restrictive respiratory failure: an observational prospective study. Respir Res.

[17] Vandenbroucke JP, von Elm E, Altman DG, Gøtzsche PC, Mulrow CD, Pocock SJ (2007). Strengthening the reporting of observational studies in epidemiology (STROBE): explanation and elaboration. Ann Intern Med.

[18] Downes JJ, Vidyasagar D, Morrow GM, Boggs TR (1970). Respiratory distress syndrome of newborn infants: I New clinical scoring system (RDS score) with acid-base and blood-gas correlations. Clin Pediatr.

[19] Keleş E, Gebeşçe A, Demirdöven M, Yazgan H, Baştürk B, Tonbul A (2016). The effects of inhaled β-adrenergic agonists in transient tachypnea of the newborn. Global Pedia Health.

[20] Singh Y, Tissot C, Fraga MV, Yousef N, Cortes RG, Lopez J (2020). International evidence-based guidelines on point of care ultrasound (POCUS) for critically ill neonates and children issued by the POCUS Working Group of the European Society of Paediatric and Neonatal Intensive Care (ESPNIC). Crit Care.

[21] Brat R, Yousef N, Klifa R, Reynaud S, Shankar Aguilera S, De Luca D (2015). Lung ultrasonography score to evaluate oxygenation and surfactant need in neonates treated with continuous positive airway pressure. JAMA Pediatr.

[22] Yoon SJ, Han JH, Cho KH, Park J, Lee SM, Park MS (2022). Tools for assessing lung fluid in neonates with respiratory distress. BMC Pediatr.

[23] O'Neill R, Dempsey EM, Garvey AA, Schwarz CE (2021). Non-invasive cardiac output monitoring in neonates. Front Pediatr.

[24] Raimondi F, Yousef N, Rodriguez Fanjul J, De Luca D, Corsini I, Shankar-Aguilera S (2019). A multicenter lung ultrasound study on transient tachypnea of the neonate. Neonatology.

[25] Saadoun D, Doya L, Dayoub A, Jouni O (2021). Transient tachypnea of the newborn and the use of prophylactic antibiotics. Am J Pedia.

[26] Corsini I, Parri N, Ficial B, Dani C (2020). Lung ultrasound in the neonatal intensive care unit: review of the literature and future perspectives. Pediatr Pulmonol.

[27] Derbent A, Tatli MM, Duran M, Tonbul A, Kafali H, Akyol M (2011). Transient tachypnea of the newborn: effects of labor and delivery type in term and preterm pregnancies. Arch Gynecol Obs.

[28] Kasap B, Duman N, Özer E, Tatli M, Kumral A, Özkan H (2008). Transient tachypnea of the newborn: predictive factor for prolonged tachypnea. Pedia Int.

[29] Bassiouny MR, Abdelhady SE, Sobh A (2022). Thoracic fluid content in neonates presented with respiratory distress as a predictive tool for transient tachypnea of newborn. Am J Perinatol.

[30] Pezza L, Sartorius V, Loi B, Regiroli G, Centorrino R, Lanciotti L (2023). Evolution of ultrasound-assessed lung aeration and gas exchange in respiratory distress syndrome and transient tachypnea of the neonate. J Pediatr.

[31] Li CS, Chu SM, Lien R, Mok TY, Hsu KH, Lai SH (2021). Prospective investigation of serial ultrasound for transient tachypnea of the newborn. Pediatr Neonatol.

[32] He L, Sun Y, Sheng W, Yao Q (2021). Diagnostic performance of lung ultrasound for transient tachypnea of the newborn: a meta-analysis. PLoS ONE.

[33] Gunes AO, Karadag N, Cakir H, Toptan HH, Karatekin G (2022). The associations between lung ultrasonography scores in the first day of life and clinical outcomes. J Ultrasound Med.

